# 7,7-Bis(3-Indolyl)-*p*-Cresol, a Metabolite from Marine-Derived Bacterium *Vibrio* spp. DJA11, Suppresses the Proliferation and Motility of Prostate Cancer Cells

**DOI:** 10.4014/jmb.2502.02035

**Published:** 2025-05-15

**Authors:** Sultan Pulat, Eun-Young Lee, Grace Choi, Yoon-Hee Jung, Sang-Jip Nam, Hangun Kim

**Affiliations:** 1 College of Pharmacy and Research Institute of Life and Pharmaceutical Sciences, Sunchon National University, 255 Jungangno, Sunchon, Jeonnam 57922, Republic of Korea; 2 Department of Chemistry and Nanoscience, Ewha Womans University, Seoul 03760, Republic of Korea; 3 Graduate Program in Innovative Biomaterials Convergence, Ewha Womans University, Seoul 03760, Republic of Korea; 4 Department of Biomaterial Research, National Marine Biodiversity Institute of Korea, Seocheon 33662, Republic of Korea

**Keywords:** Marine natural product, *Vibrio* spp., prostate cancer, proliferation, motility, AKT/mTOR

## Abstract

Bacteria such as *Vibrio* spp. in the marine environment can produce secondary metabolites which have significant potential applications in pharmaceuticals. In a study to discover bioactive secondary metabolites from marine *Vibrio* spp., the strain DJA11 was encountered. HPLC/UV-guided isolation of the crude extract from this strain has led to the discovery of compound 1. Prostate cancer (PCa) is one of the biggest worldwide health issues because of its high diagnosis. CWR22Rv1 (22Rv1) is mutated in WT p53 and AR, C4-2 is derived from androgen-dependent human LNCaP and PC-3 is an androgen-independent cancer cell type. It was found that compound 1 exhibited no significant cytotoxicity at concentrations below 50 μM to human PCa cells, including 22Rv1, C4-2, and PC-3, like normal cell HEK293T. In addition, we presented that 1 inhibited the invasiveness and proliferation of 22Rv1, PC-3, and C4-2 cells by suppressing the activation of p-AKT, p-mTOR, p-STAT3, HSP90, and HSP70. Moreover, treatment with 1 decreased the mRNA expression level of ErbB4, PDK1, STAT3, HSP70, and HSP90 in some PCa cells. Therefore, compound 1 may have therapeutic potential in PCa due to its role in suppressing cancer proliferation and metastasis.

## Introduction

Marine environments are an untapped reservoir of diverse microorganisms, including bacteria, fungi, and archaea, which are known to produce unique bioactive compounds [1, 2]. Among these, species of the genus *Vibrio* have attracted particular attention due to their remarkable adaptability to diverse and often extreme marine habitats, which promotes the synthesis of complex secondary metabolites [3, 4] *Vibrio* spp. are Gram-negative, rod-shaped bacteria that inhabit a wide range of aquatic ecosystems, including freshwater systems, estuarine environments, and marine habitats [5]. This ecological versatility facilitates their production of a diverse array of secondary metabolites. Beyond their ecological role as a vital component of the marine food web, *Vibrio* spp. is a valuable source of bioactive molecules with significant potential applications in pharmaceuticals, nutraceuticals, and various other industries.

The unique metabolic versatility of marine-derived *Vibrio* spp. is demonstrated by their capacity to produce structurally diverse compounds, including alkaloids [6], polyketides [7], nonribosomal peptides [8], and others, many of which exhibit potent antibacterial [9], antimicrobial [10], antifungal, anticancer, and antioxidant activities [4]. As a part of collaborative study to discover bioactive secondary metabolites from marine *Vibrio* spp., the strain DJA11 was encountered. HPLC/UV-guided isolation of the crude extract from this strain has led to the discovery of compound **1**.

HPLC-UV-guided fractionation of the crude extract from the DJA11 strain, isolated from *Ulva australis*, has led to the identification of 7,7-bis(3-indolyl)-*p*-cresol (**1**). The chemical structure was elucidated based on detailed spectroscopic data, analyses, including MS, UV, and NMR, and further validated through a comparative assessment with the previously reported structure.

Prostate cancer (PCa) is one of the most commonly diagnosed cancers worldwide, it is the third most common cancer type in men [11, 12]. The number of PCa cases increases each year in the world, World Health Organization (WHO) statistics show PCa cases rose from 1.1 million in 2012 to over 1.4 million in 2020 [13].

In this research, three different prostatic cancer cells were selected, the normal cell was chosen for comparison of the effect of PCa cells and normal cells; CWR22Rv1 (22Rv1), C4-2, PC-3, and HEK293T (human embryonic kidney) cells. 22Rv1 is an androgen-independent human prostate carcinoma cell, which has a mutation in WT p53 and AR (T877A) [14]. C4-2 prostatic cancer cells are derived from androgen-dependent human LNCaP [15]. PC-3 cells cannot produce prostate-specific antigen (PSA) and are androgen-independent which distinguishes PC-3 cells from 22Rv1 and C4-2 [16]. The three different PCa cells, classified as AR-dependent or AR-independent, allowed us to assess the anti-cancer effect of compound **1** across various PCa phenotypes and determine whether **1** suppresses the PCa metastasis and proliferation via AR or other signalings.

PCa is associated with over-activating AKT-mTOR signal pathways like many human cancers. mTOR has two distinct complexes which are mTORC1 and mTORC2. mTORC2 serves as an upstream kinase in the AKT-mTOR signal pathway [17, 18]. Heat shock proteins 70 (HSP70) can control protein homeostasis, and signal transduction. Overexpression of HSP70 has negative outcomes like promoting cell viability, cancer metastasis, and inhibiting chemotherapy efficiency [19]. PI3K/AKT/GSK3β signal pathway induced overexpression of HSF1 which has a crucial role in HSP70 protein synthesis [20]. Everolimus is used as an mTOR inhibitor in the suppression of cancer development, however, its clinical application is limited because of side effects. Everolimus suppresses PI3K/AKT/mTOR by blocking the mTOR which promotes cancer cell metastasis and proliferation. Although the anticancer effect of mTOR inhibitor, everolimus, was reported, a previous study demonstrated that it promotes treatment-related death in cancer patients. Therefore, investigating a new compound that inhibits mTOR and AKT activity is crucial for cancer treatment [21].

As a transcription factor, STAT3 is translocated to the nucleus and binds to the specific target gene on DNA therefore, it can control gene transcription. HSP90 supports the STAT3 activation by enhancing the phosphorylation, and translocation of STAT3 to bind the target gene [22].

The androgen receptor (AR) is a kind of ligand-dependent nuclear transcription factor that can bind to testosterone or dihydrotestosterone (DHT), over-activation of AR causes the proliferation and survival of PCa cells [23, 24]. Heat shock proteins 90 (HSP90) and HSP70 interact with AR thereby enhancing the stability of the AR [25]. One of the most popular AR inhibitors is enzalutamide, which prevents AR from interacting with DNA. However, its clinical application is limited because long-term use increases the risk of cardiovascular disease due to side effects. Thereby, the investigation of the alternative compounds that suppress AR in cancer treatment is important for effective cancer treatments [26].

Previous studies have demonstrated that **1** has an anticancer effect on the metastatic potential of hormone-dependent and hormone-independent breast cancer and an anti-proliferative effect on lung cancer [27, 28]. However, the anticancer effect of **1** in PCa cells has not been thoroughly investigated. In this study, we aimed to investigate the effects of **1** on the proliferation and metastasis of PCa cells; CWR22Rv1 (22Rv1), C4-2, and PC-3 through affect key signaling, including p-AKT, p-mTOR, p-STAT3, HSP90 HSP70 ErbB4, and PDK1.

The effect of **1** on cell viability was assessed by MTT assay, while its on PCa cell invasion ability was evaluated by trans well invasion. The effect of **1** on proliferation was tested by clonogenic assay. These phenotypic assay results were confirmed with the protein level by western blot and mRNA expression levels by quantitative real-time PCR.

## Experimental Section

### General Experimental Procedures

NMR spectra were acquired using an Agilent 400-MR DD2 spectrometer (Agilent Technologies, USA) at 400 MHz and 100 MHz for ^1^H and ^13^C NMR, respectively, containing Me_4_Si as internal standard and using solvent DMSO-*d_6_* (Cambridge Isotope Laboratories (CIL), Inc., USA) equipped at Ewha Drug Development Research Core Center. Low-resolution LC-MS measurements were performed using the Agilent Technology 1260 quadrupole (Agilent Technologies) and Waters Alliance Micromass ZQ LC-MS system (Waters Corp., USA) using reversed-phase column (Phenomenex Luna C18 (2) 100 Å, 100 mm × 4.6 mm, 5 mm) (Phenomenex, USA) at a flow rate 1.0 ml/min at the National Research Facilities and Equipment Center (NanoBioEnergy Materials Center) at Ewha Womans University.

### Isolation and Identification of the Strain

We isolated the DJA11 strain from *Ulva australis* collected in Dangjin, Chungcheongnam-do, South Korea (37°1' 3.8'' N 126° 27' 42.3'' E). In detail, the collected *Ulva australis* was first throughly washed with sterilized seawater to remove any debris and contaminations. It was then finely chopped using sterilized scissors and ground into a paste with a sterilized mortar and pestle inside a clean bench to ensure aseptic conditions. The resulting homogenate was suspended in sterilized seawater and diluted to a 1:20 ratio. This diluted suspension was spread onto Marine Broth 2216 (Merck Group, Germany) agar plates, which were incubated to promote microbial growth. Through repeated streaking and subculturing, the pure strain DJA11 was successfully isolated.

For species identification of the DJA11 strain, 1 ml of the culture grown in marine broth liquid medium at 27°C for 4 days was harvested. Genomic DNA was then extracted using a Tissue Genomic DNA Kit (Cosmogenetech Co. Ltd., Republic of Korea), following the manufacturer’s protocol. For 16S rRNA gene amplification to identify the DJA11 strain, PCR was performed using the primers 27F and 1492R. The PCR product was purified using a PCR purification Kit (Cosmogenetech Co. Ltd.) and sequenced using a capillary electrophoresis system (Applied Biosystems 3730XL). The resulting 16S rRNA gene sequence obtained from the DJA11 strain was compared to previously reported sequences in the GenBank/EMBL/DDBJ database using BLAST search. As a result, the 16S rRNA gene sequence of the DJA11 strain showed 99.9% similarity to that of *Vibrio kanaloae* LMG 20539, indicating that the DJA11 strain belongs to the *Vibrio* genus. The DJA11 strain was deposited in the Korean Collection for Type Culture (KCTC) on August 20, 2024, and was assigned the accession number KCTC 16007BP.

### Cultivation and Extraction

The strain DJA11 was cultured in 80 × 2.5 L Ultra Yield flasks each containing 1 L of medium (37.4 g/l marine broth 2216 (BD Difco^TM^, USA) dissolved in distilled H_2_O) at 30°C with constant shaking at 130 rpm. After 6 days, the broth was extracted with EtOAc (80 L overall), and the EtOAc-soluble fraction was dried *in vacuo* to yield 1.30 g of organic extract.

### Isolation of the Compound 1

The organic extract (1.30 g) was fractionated by C18 resin eluted with 100 ml H_2_O/CH_3_OH (80/20, 60/40, 50/ 50, 40/60, 30/70, 20/80, and 0/100) to obtain seven fractions (F1–F7). Fraction 5 (F5) was further purified by reversed-phase HPLC (Phenomenex Luna C-18 (2), 250 × 100 mm, 2.0 ml/min, 5 mm, 100 Å, UV = 210 nm) under an isocratic condition with 48% aqueous CH_3_CN (0.01% trifluoroacetic acid in H_2_O) to yield 7,7-bis(3-indolyl)-*p*-cresol (**1**, 3.4 mg, *t*_R_ = 45.5 min).

*7,7-Bis(3-indolyl)*-*p*-*cresol* *(**1**)*: yellow solid; ^1^H NMR (400 MHz, DMSO-*d_6_*): δ_H_ 10.75 (s, NH), 9.12 (s, OH), 7.32 (2H, d, *J* = 8.0 Hz, H-7', 7''), 7.25 (2H, d, *J* = 8.0 Hz, H-4', 4''), 7.13 (2H, d, *J* = 8.5 Hz, H-3, 5), 7.01 (2H, t, *J* = 8.6 Hz, H-6', 6''), 6.84 (2H, t, *J* = 8.6 Hz, H-5', 5''), 6.77 (2H, d, *J* = 2.0 Hz, H-2', 2''), 6.65 (2H, d, *J* = 8.5 Hz, H-2, 6) , 5.69 (^1^H, s, H-7); ^13^C NMR (100 MHz, DMSO-*d_6_*): δc 155.2 (C-1), 136.6 (C-7a', 7a''), 135.2 (C-4), 129.1 (C-3, 5), 126.6 (C-3a', 3a''), 123.3 (C-2', 2''), 120.7 (C-6', 6''), 119.2 (C-4', 4''), 118.6 (C-3', 3''), 118.0 (C-5', 5''), 114.7 (C-2, 6), 111.4 (C-7', 7''), 38.8 (C-7); LR-ESI-MS (positive) *m/z*: 338.2 [M + H]^+^.

### Cell Culture

PCa cell lines 22Rv1, C4-2, PC-3, and HEK293T were cultured in Roswell Park Memorial Institute (RPMI) 1640 Medium or DMEM (Dulbecco's Modified Eagle Medium (Gen Depot, USA), supplemented with 10% FBS and 1%penicillin-streptomycin solution in a humid environment with 5% CO_2_ [29].

### Methyl Thiazolyl Tetrazolium (MTT) Assay

PCa cell lines 22Rv1, C4-2, PC-3, and HEK293T were seeded on 96-well plates for grown overnight, and then treated with DMSO (Sigma-Aldrich, USA) or 10, 25, 50, and 100 μM of compound **1** for 48 h. The cells were lysed with 150 μl of DMSO after 4 h of incubation with MTT in 5% CO_2_ at 37°C. The absorbance was measured at 540 nm using a microplate reader and analyzed with Gen 5 (2.03.1; BioTek, USA) [30].

### Invasion Assay

The 22Rv1, PC-3 (3 × 10^5^), and C4-2 (2.5 × 10^5^) cells were seeded in a culture medium containing 0.2% bovine serum albumin (BSA) and incubated with 5 and 10 μM of compound **1** or DMSO control for 24 h in polycarbonate membranes with 8 μm pores coated with 1% gelatin trans well chambers (Corning, USA). The lower chamber was filled with 600 μl RPMI containing 0.2% BSA and 6 μg/ml fibronectin (EMD Millipore Corp., USA) as a chemoattractant for 24 h incubation. Diff-Quik kit (Sysmex, Japan) was used for fixed and staining of invading cells. The number of cells was quantified using a Nikon Eclipse 400 fluorescence microscope (Nikon Instech, Co. Ltd., Japan) and i-Solution FL Auto Software (IMT i-Solution Inc., Canada; five fields/chamber) [31].

### Clonogenic Assay

22Rv1, PC-3, and C4-2 were seeded at a density of 0.5–1×10^3^ cells/well in RPMI medium and incubated to encourage attachment and then treated with DMSO, 5 μM, and 10 μM of **1**. Cells were stained with 0.01% crystal violet after seeding at 14 days, colony areas were measured by the IMT iSolutionFL software [32].

### Quantitative Real-Time PCR

Briefly, the total RNA of 22Rv1, PC-3, and C4-2 cells were isolated by using RNAiso Plus (Takara, Japan). Moloney murine leukemia virus reverse transcriptase (Invitrogen, USA) was used to convert RNA to cDNA, and relative gene expression was analyzed by the dye SYBR Green (Enzynomics, Republic of Korea). Further, the qRT-PCR reaction and analysis were performed using CFX (Bio-Rad, USA) [33]. The primers used for real-time PCR were as follows: GAPDH (forward) 5'-ATCACCATCTTCCAGGAGCGA-3'; and Reverse 5’-AGTTGTCATGGA TGACCTTGGC-3'; HSP70 (forward) 5'-TTGCGCAAGGCTCGGTACTG-3' and TTTTCTGCTGGTGTCTGCTG; HSP90 (forward) 5'- TTCTGCTTATTTGGTTGC-3' and 5'-AACTTTTGTTCCACGACC-3'; PDK1 (forward) 5'-TTACGGATTGCCCATATCACG-3' and 5'-CCCGGTCACTCATCTTCACAGT-3', ERBB4 (forward) 5'-ATGAAGCCGGCGACAGGACT-3' and5'-TTGCGCAAGGCTCGGTACTG-3'. GAPDH primer was used as a housekeeping gene for normalization.

### Molecular Docking

The PDB structures of AKT1 (2XJX), mTOR (4JT6), and AR (2Q7I) were obtained from the RCSB PDB database, while the chemical structures of 1 (2940609) and testosterone (13475125) were extracted from the PubChem database for the docking analysis. Water molecules and the crystal ligand were removed from the protein-ligand complex before importing it into AutoDockTools (1.5.7) for docking. Two-dimensional (2D) and three-dimensional (3D) diagrams of intermolecular interactions were analyzed using Discovery Studio software.

### Western Blots

22Rv1, PC-3, and C4-2 cells were treated with 5 or 10 μM of **1** for 24 h. 25 μg of lysed protein was separated by sodium dodecyl sulfate-polyacrylamide gel electrophoresis. Multigauge 3.029 was used to measure bands for each sample and the results were normalized to that of α-tubulin or glyceraldehyde 3-phosphate dehydrogenase (GAPDH). α-tubulin and GAPDH were used as a loading control. Values were expressed as densitometric units, corresponding to the signal intensity [34].

## Results and Discussion

### Identification of Compound 1

**Fig. 1:**
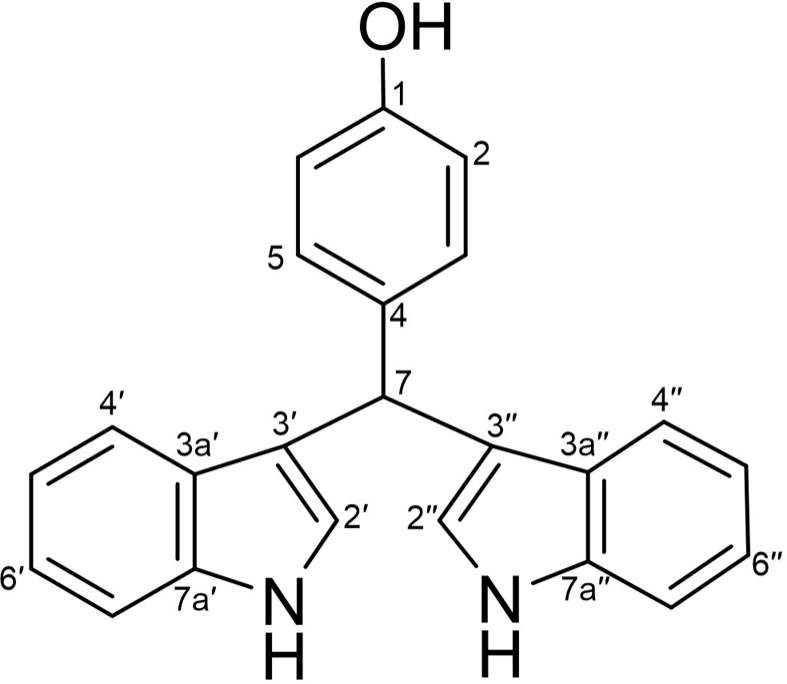
Chemical structure of 1 (7,7-bis(3-indolyl)-*p*-cresol).

Compound **1** was isolated as yellow solid, and LR-ESI-MS revealed an ionic peak at *m/z* 338.2 [M+H]^+^. The ^1^H NMR spectrum of **1** showed two exchangeable protons at 1-OH (δ_H_ 9.12, s), and NH (δ_H_ 10.75, s), three methine proton at H-7 (δ_H_ 5.69, s, ^1^H), H-2' and H-2'' (δ_H_ 6.77, d, *J* = 2.0 Hz, 2H), and fourteen aromatic protons at H-2 and H-6 (δ_H_ 6.65, d, *J* = 8.5 Hz, 2H), H-3 and H-5 (δ_H_ 7.13, d, *J* = 8.5 Hz, 2H), H-5' and H-5'' (δ_H_ 6.84, t, *J* = 8.6 Hz, 2H), H-6' and H-6'' (δ_H_ 7.01, t, *J* = 8.6 Hz, 2H), H-4' and H-4'' (δ_H_ 7.25, d, *J* = 8.0 Hz, 2H), and H-7' and H-7'' (δ_H_ 7.32, d, *J* = 8.0 Hz, 2H) (Fig. S1). The ^13^C NMR spectrum of **1** displayed eighteen carbons at C-1 (δc 155.2), C-2 and 6 (δc 114.7), C-3 and C-5 (δc 129.1), C-4 (δc 135.2), C-4' and 4'' (δc 119.2), C-5' and 5'' (δc 118.0), C-6' and 6'' (δc 120.7), C-7' and 7'' (δc 111.4), C-3a' and 3a'' (δc 126.6), C-7a' and 7a'' (δc 136.6), two indole moeity carbons at C-2' and C-2'' (δc 123.3), and C-3' and C-3'' (δc 118.6), and one methine carbon at C-7 (δc 38.8) (Fig. S2). Based on the comparison of the ^1^H NMR and ^13^C NMR data of **1** with those reported in the literature, compound **1** was identified as 7,7-bis(3-indolyl)-*p*-cresol (Fig. 1).

### Effect of 1 on the Cell Viability of Prostate Cancer (PCa) Cell Lines

**Fig. 2:**
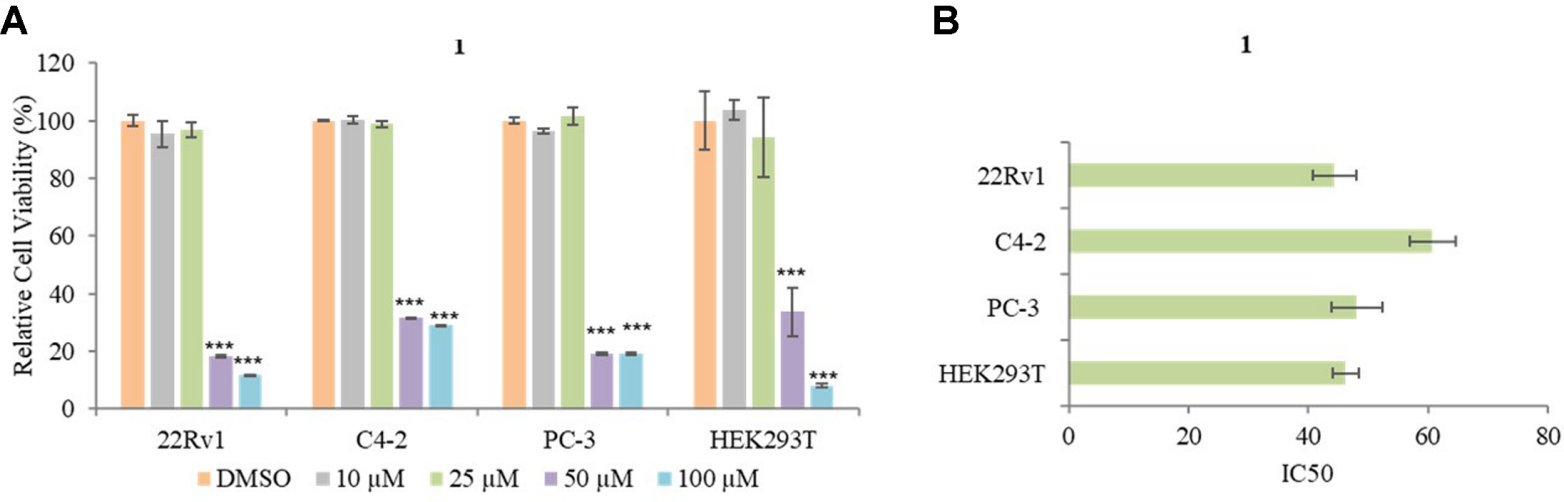
The effect of 1 on the cell viability of PCa cell lines. (**A**) Relative cell viability of 22Rv1, C4-2, PC-3, and HEK293T were measured by MTT after treatment of 10, 25, 50, and 100 μM of 1. (**B**) IC_50_ values of 1 against the 22Rv1, C4-2, PC-3, and HEK293T. Data are presented as mean ± SD, *n* = 3. ***p < 0.001.

MTT assay was conducted to measure the effect **1** on PCa and HEK293T cell viability. 22Rv1, PC-3, C4-2, and HEK293T were seeded on a 96-well plate for grown overnight, and then treated with 10, 25, 50, and 100 μM of **1** for 48 h. For comparison of the effect of 1, three different PCa cells were selected in the study, as well as a human embryonic kidney as a control. Treatment with 10 and 25 μM of compound **1** did not inhibit the cell viability of PCa and HEK293T while 50 and 100 μM of compound **1** significantly inhibited the cell viability (Fig. 2A). The IC_50_ of **1** was 44.3 μM in 22Rv1, 60.7 μM in PC-3, 48 μM in C4-2, and 46.2 μM in HEK293T (Fig. 2B). Thus, these observations demonstrate that a high concentration of compound **1** has a strong bioactivity for reducing PCa and normal cell viability. A nontoxic concentration of **1** (5 and 10 μM) was used to show the effect of **1** on PCa cell proliferation and metastasis for further analysis.

### The Effects of 1 on the Motility and Proliferation of Prostate Cancer (PCa) Cell Lines

**Fig. 3:**
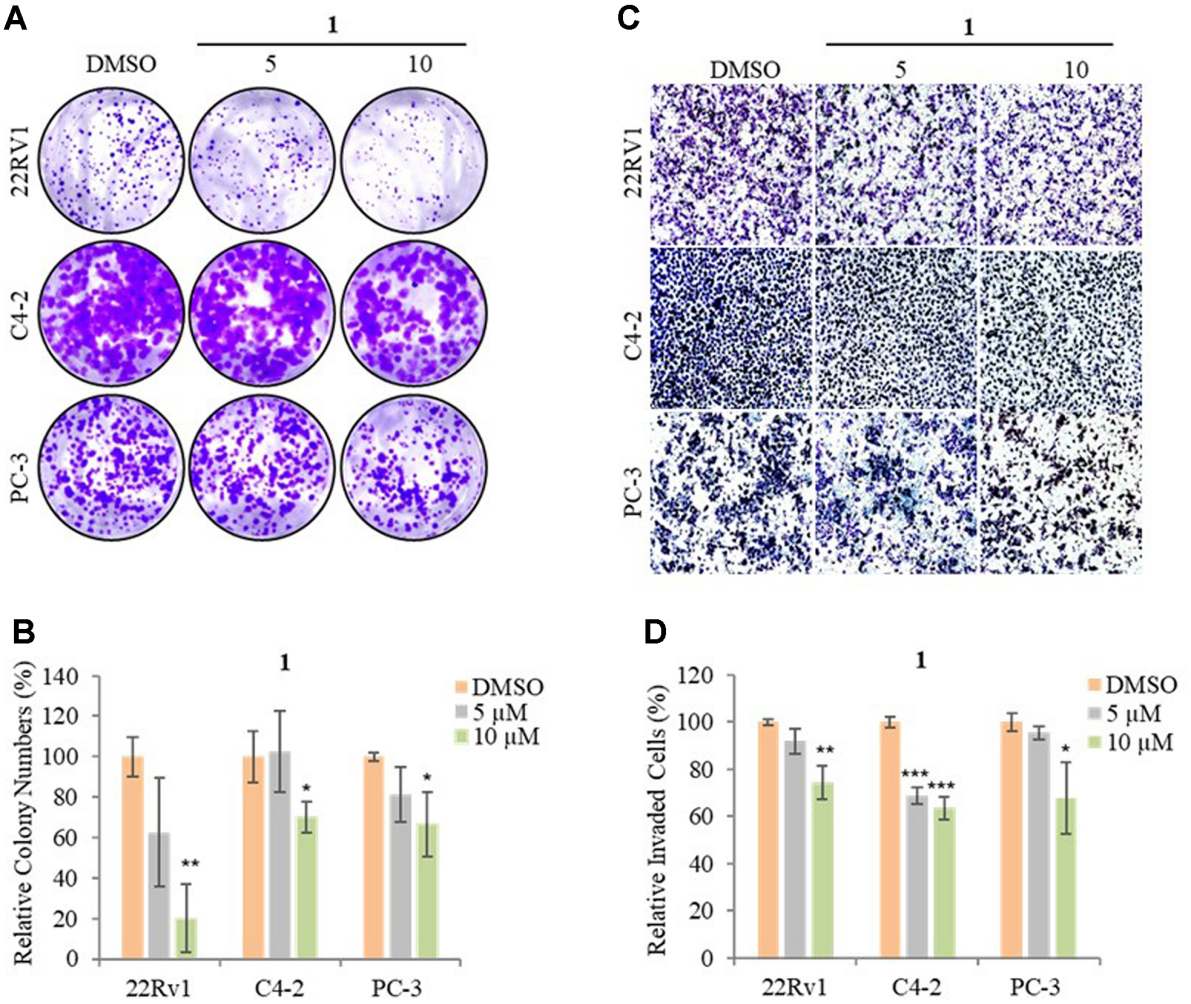
The effects of 1 on the motility and proliferation of PCa cell lines. (**A**) Representative images of each insertion in the clonogenic assay. (**B**) The relative percentage of colony numbers on 22Rv1, C4-2, and PC-3 cells. (**C**) Representative images of each insertion in the invasion assay. (**D**) Relative percentage of invaded 22Rv1, C4-2, and PC-3 cells. Data are presented as mean ± SD, *p < 0.05; **p < 0.01; ***p < 0.001.

The PCa cells have longer life spans and higher proliferation capacity than normal cells therefore, suppressing proliferation is a promising strategy to reduce cancer mortality [35]. Hence, a clonogenic assay was conducted to show whether treatment with 5 and 10 μM of **1** affects the proliferation of 22Rv1, PC-3, and C4-2 cells. As shown in Fig. 3A, **1** significantly reduced the number of colonies by ~80%, ~30%, and ~35% at 10 μM on 22Rv1, PC-3, and C4-2, indicating that cell proliferation was inhibited. Taken together, these results indicated that 10 μM of **1** inhibited cell proliferation in 22Rv1, C4-2, and PC-3 cells.

Metastasis is the spreading of cancer cells to the secondary space of the body from where it started which is one of the main reasons for mortality in PCa patients [36]. To elucidate whether 5 and 10 μM of **1** affect 22Rv1, PC-3, and C4-2 cell motility, an invasion assay was conducted. Treatment with 5 μM of **1** did not decrease the invaded ability of 22Rv1, C4-2, and PC-3 while 10 μM of **1** significantly inhibited the invaded ability of all three (Fig. 3C and 3D). We show that a nontoxic concentration of **1** (10 μM) suppressed the invasion ability of PCa cells.

### The Effects of 1 on the Protein Level of p-AKT, p-mTOR, p-STAT3, HSP90, and HSP70 in Prostate Cancer (PCa)

**Fig. 4:**
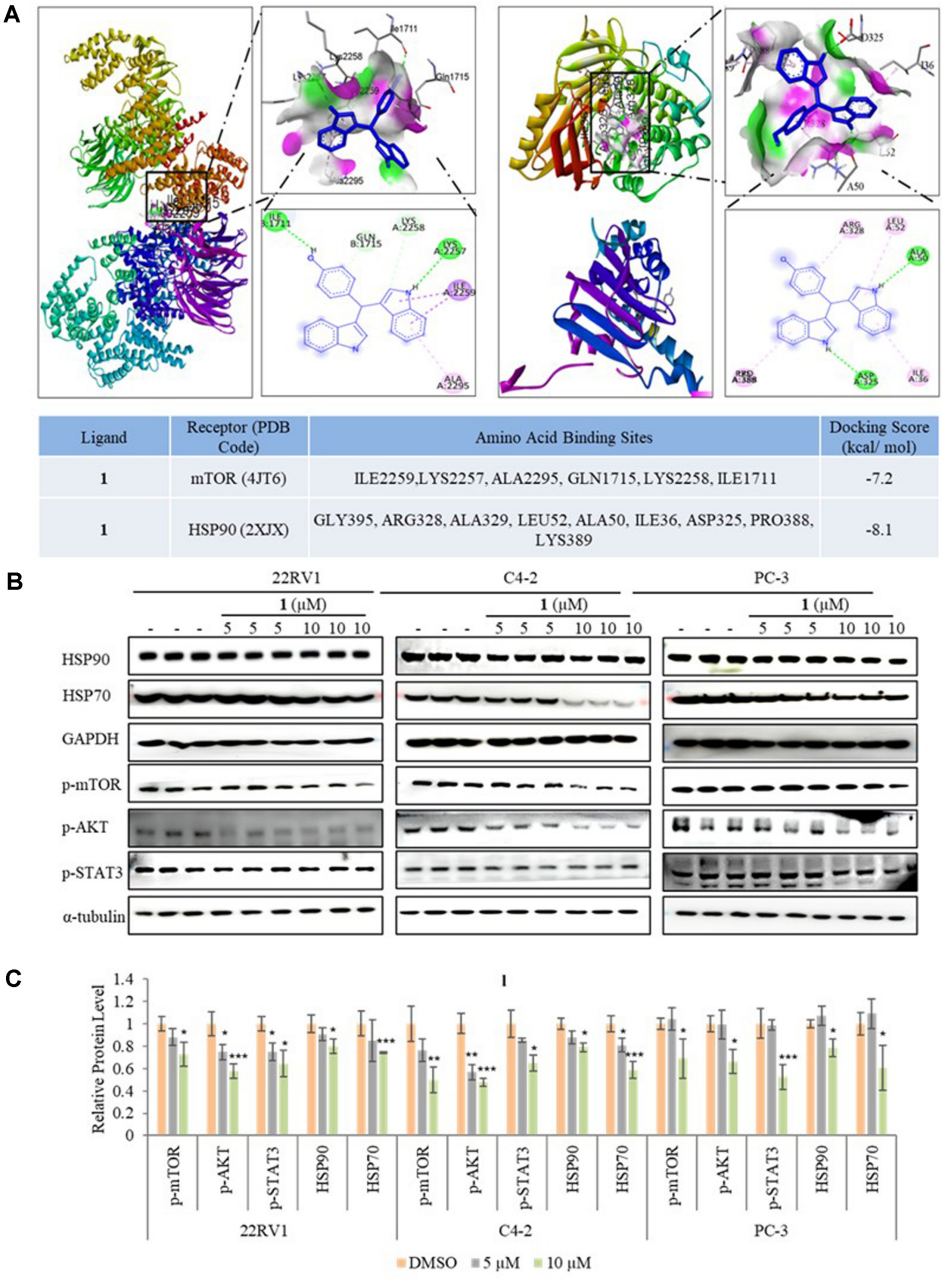
Three-dimensional and two-dimensional structural interactions of important target receptors with 1, pink indicating the donor and green indicating the acceptor. (**A**) Interaction of mTOR with **1** and interaction of HSP90 with **1**. (**B**) Western blot analysis of HSP90, HSP70, p-AKT, p- mTOR, and p-STAT3. (**C**) Relative protein levels of HSP90, HSP70, p-AKT, p-mTOR, and p-STAT3. Data are presented as mean ± SD, *n* = 3. *p < 0.05; **p < 0.01; ***p < 0.001.

*In silico*, the intracellular target for **1** was identified by Swiss Target Prediction. **1**’s smile code was used for the target prediction species, which detected 100 different targets. Next, docking scores were shown based on the Swiss Target web server offers. As in Fig. 4, **1** docking scores for mTOR, and HSP90, range from -7.4 to -8.1 kcal/mol through a conventional hydrogen bond, carbon-hydrogen bond, unfavorable donor-donor, pi-cation, pi-donor hydrogen, pi-sigma, pi-pi stacked, and pi-alkyl interactions.

Heat shock proteins are involved in mTOR signaling by affecting the proper protein folding [37, 38]. STAT3 plays an important role in cancer proliferation and metastasis as a transcription factor, and STAT3 is one of the downstream targets of the AKT/mTOR pathway [39, 40]. To determine whether the inhibition of motility in PCa cells in the presence of nontoxic concentrations of **1** involves AKT, mTOR, STAT3, HSP90, and HSP70, the protein level of these proteins was determined by western blot.

Compound **1** at 10 μM significantly decreased the protein level of p-AKT, p-mTOR, and p-STAT3 in three PCa cells. In addition, **1** also reduced the protein level of HSP90 and HSP70 (Fig. 4B and 4C). These results show that **1** inhibited the invasiveness and proliferation of 22Rv1, PC-3, and C4-2 cells by suppressing the activation of p-AKT, p-mTOR, p-STAT3, HSP90, and HSP70, suggesting that **1** has therapeutic potential in PCa.

### The Effect of 1 on mRNA Expression of ErbB4, PDK1, STAT3, HSP70, and HSP90 in Prostate Cancer (PCa)

**Fig. 5:**
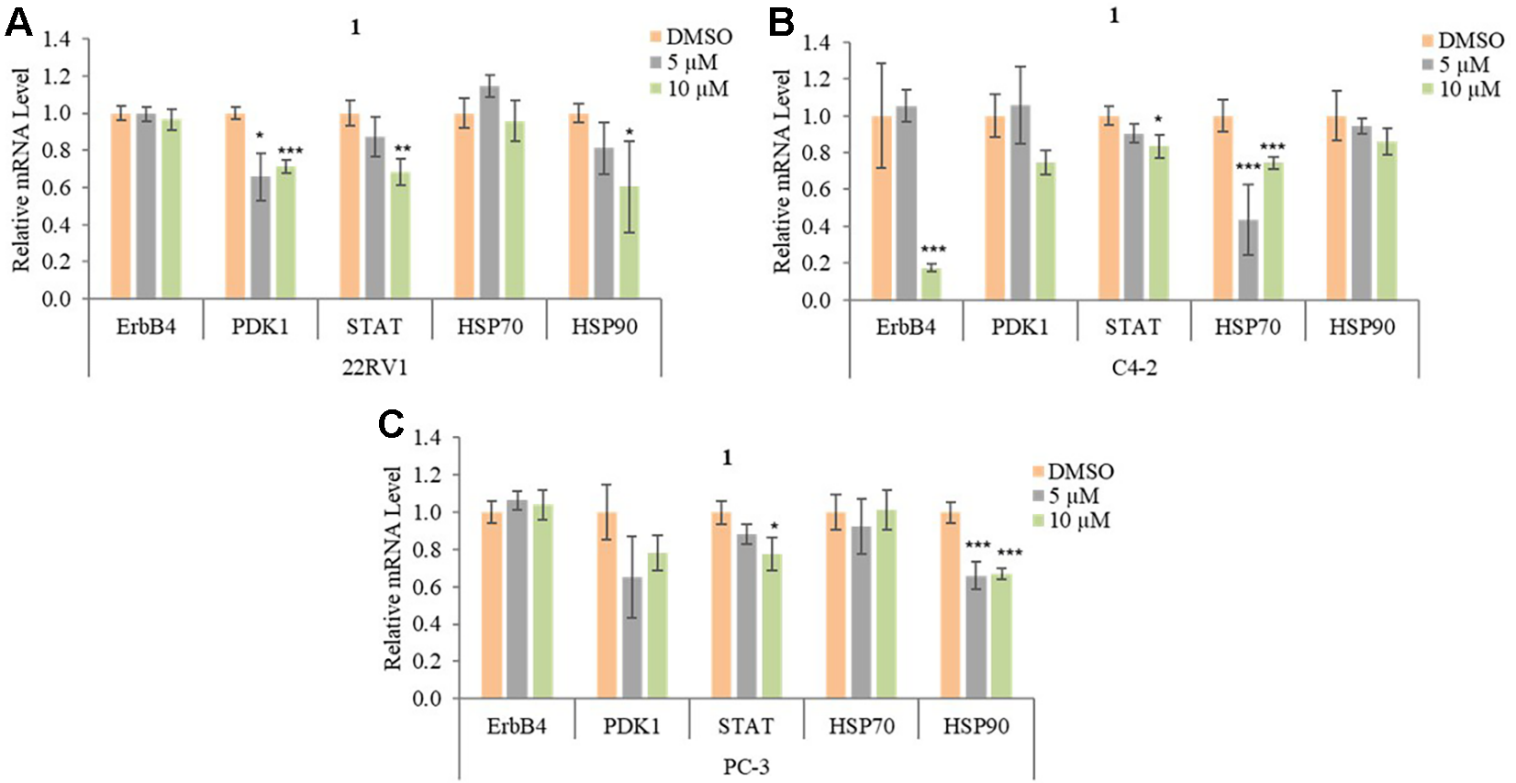
Effect of 1 on the mRNA expression of ErbB4, PDK1, STAT3, HSP70, and HSP90. (**A**) Relative mRNA levels of of ErbB4, PDK1, STAT3, HSP70, and HSP90 in 22RV1. (**B**) Relative mRNA levels of ErbB4, PDK1, STAT3, HSP70, and HSP90 in C4-2. (**C**) The mRNA expression levels of ErbB4, PDK1, STAT3, HSP70, and HSP90 in PC-3. Data are presented as mean ± SD, *n* = 3. *p < 0.05; **p < 0.01; ***p < 0.001.

ErbB4 is involved in the development of cancer by different signal pathways and mechanisms which include modifying the PI3K/AKT/mTOR pathways by promoting gene transcription by STAT3 [41]. Phosphorylation of AKT is increased by different kinase types like PDK1 and mTORC2, and overexpression of AKT promotes cancer cell viability, proliferation, and tumor growth. In addition, PDK1 has a significant role in activating downstream targets such as AKT in PCa [42]. This information suggests that ErbB4 and PDK1 can be therapeutic targets and may modulate sensitivity to cancer therapies. Treatment with 10 μM of **1** significantly inhibited the mRNA expression level of ErbB4, STAT3, and HSP70 while 10 μM of **1** did not change the mRNA expression level of PDK1, HSP90 in C4-2. In addition, we show that 10 μM of **1** decreased the mRNA expression level of PDK1, STAT3, and HSP70 whereas **1** did not affect the mRNA expression level of ErbB4 and HSP90 in PC-3 (Fig. 5). Taken together, these results indicated that compound **1** inhibits different oncogenes expression levels across three types of PCa cells through various suppressed mechanisms.

### The Effects of 1 on AR in Prostate Cancer (PCa)

**Fig. 6:**
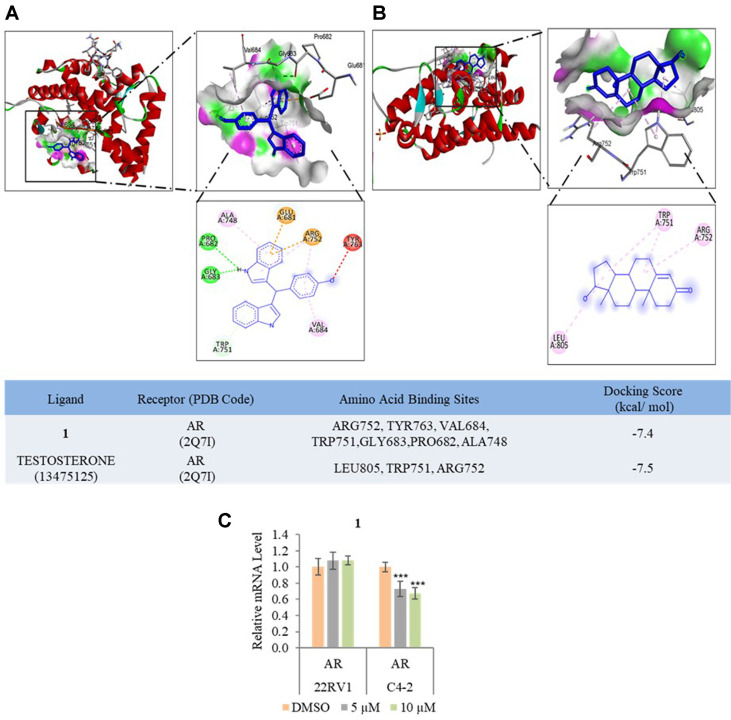
The effect of 1 on AR. (**A**) Interaction of AR with 1. (**B**) Interaction of AR with testosterone. (**C**) Effect of 1 on mRNA expression level of AR in 22Rv1, and C4-2 cells. Data are presented as mean ± SD, *n* = 3. ***p < 0.001.

AR is a steroid hormone receptor that binds testosterone and DHT, thereby promoting PCa progression [43]. In silico, the intracellular target for **1** was identified by Swiss Target Prediction, docking scores were shown based on the Swiss Target web server offers. As in Fig. 6, **1** docking scores for AR for **1** and testosterone are -7.4 and -7.5 kcal/mol through a conventional hydrogen bond, unfavorable donor-donor, Pi-cation, Pi-donor hydrogen, Pi-anion, and Pi-alkyl interactions. To elucidate whether 5 and 10 μM of **1** affect the AR on 22Rv1, and C4-2 cells, the mRNA expression level of AR was checked. Treatment with **1** decreased the mRNA expression level of AR in C4-2 cells while **1** did not affect this gene expression in 22Rv1 cells.

### Target of Compound 1 and Prostate Cancer (PCa)

**Fig. 7:**
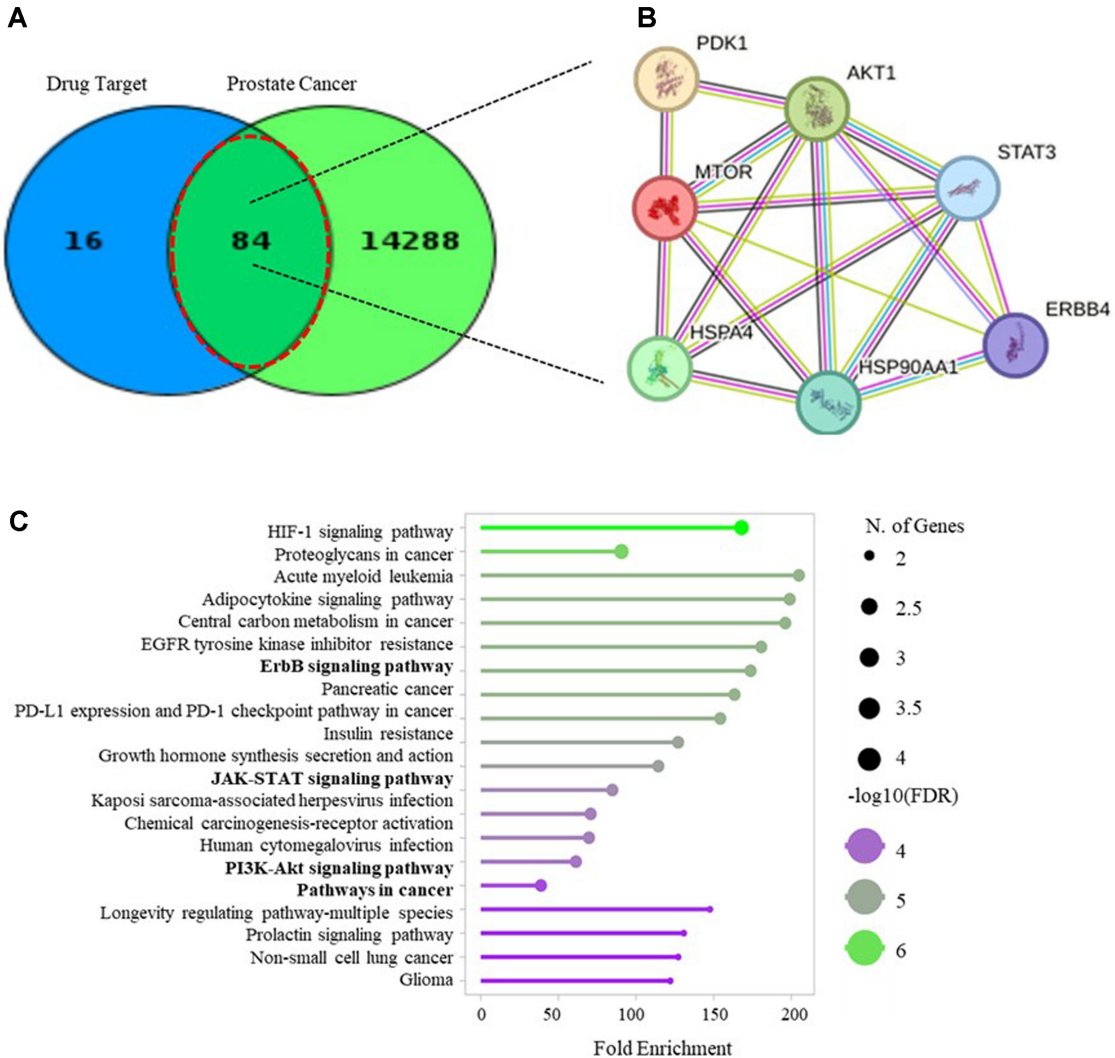
(**A**) Genes in common between PCa and 1 using the Venny. This figure compares the total number of target genes of **1** and PCa and shows the overlap between them at the intersection. (**B**) Gene network analysis by STRING. (**C**) Pathway assessment chart of the genes using the Shinny. Pathways are color-coded according to FDR.

The Venny diagram shows genes associated with PCa, identifying 100 as potential targets of **1** (Fig. 7A). Overlap between PCa’s and potential targets of **1** in the intersection includes 84 genes, mTOR, HSP90AA1, AR, and PDK1 placed in this area. We indicated that the protein-to-protein network analysis of **1** (Fig. 7B). Gene ontology analysis is shown in Fig. 7C based on AKT, mTOR, STAT3 HSP90, HSP70, ErbB4, and PDK1. The signal pathways in which we show the protein or mRNA levels, are in the first 20-fold enrichment level. These signal pathways are the ErbB signaling pathway, PD-1 checkpoint pathway, JAK-STAT signaling pathway, PI3K-AKT signaling pathway, and pathways in cancer. ErbB signaling pathway regulates cancer cell proliferation with conjugation of AKT [44]. The JAK-STAT signaling pathway is important in cancer progression high activation of this signal path promotes cell proliferation, and metastasis [45]. We show that **1** significantly inhibited the protein level of p-AKT, p-mTOR, p-STAT3 HSP90, HSP70 and mRNA expression level of ErbB4, PDK1, STAT3, HSP70, and HSP90. These are components of the ErbB, JAK-STAT, and PI3K/AKT signaling pathway.

## Discussion

Compound 1 exhibits diverse biological activities, including antimicrobial effects [46], and the ability to act as an antagonist of the orphan nuclear receptor Nur77 (NR4A1, TR3) [47]. It induces reactive oxygen species (ROS) production and ER stress in pancreatic cancer cells [48]. Moreover, it mimics Nur77 knockdown effects in non-small-cell lung cancer cells (A549, H460), resulting in the inhibition of proliferation, induction of apoptosis, and suppression of mTORC1 signaling [27, 48]. Additionally, it downregulates β1- and β3-integrin expression, impairing breast cancer cell migration [28], and decreases histone methyltransferase G9A (EHMT2) expression in alveolar rhabdomyosarcoma cells, underscoring its broad therapeutic potential [49]. However, the biological activity of **1** in PCa cells has not been explored. We aim to investigate the anticancer effect of **1** in PCa, focusing on its ability to suppress cancer proliferation and metastasis which has a promising strategy to reduce PCa mortality.

Uncontrolled growth by high stimulation of some signal pathways is the reason for PCa in the gland which is placed in the reproductive system. PCa patients often do not have clear symptoms in the cancer early stage but in later stages, symptoms can be seen [50]. The risk of developing PCa is associated with these factors; older age, race, family history, and obesity [51]. Like other kinds of cancer PCa is caused by mutations in DNA that cause cells to have a longer life span and divide faster compared to normal cells. With the high proliferation capacity, these cells start to accumulate in their primary place and then according to their capacity, these cells can migrate to their secondary place by blood vessels. All these processes occur through signal pathways that are overstimulated.

In this study, we show that a high concentration of compound **1** has a strong suppressing effect on 22Rv1, C4-2, and PC-3 cell viability. PCa cells have a longer lifespan and a higher proliferation capacity than normal cells, so suppressing proliferation is a promising strategy to reduce cancer mortality. A nontoxic concentration of **1** inhibited cell proliferation and metastasis in PCa cells. Therefore, these results demonstrated that treatment with **1** showed an anti-cancer effect and a safer treatment without its non-toxic concentrations in PCa cells. In the other analyses focused on elucidating the underlying mechanisms by which compound **1** reduces cell proliferation and metastasis.

AKT can be activated by PDK1 and mTOR, and over-activation of AKT can cause proliferation and survival of cancer cells [42]. We show treatment with **1** decreased significantly the protein level of p-AKT, and p-mTOR in three PCa cells.

Previous studies have shown that STAT3 is also an oncogenic protein that promotes primary prostate tumors and metastatic lesions in PCa [52]. In this study, we indicate that treatment with **1** suppressed the protein level and mRNA expression level of STAT3 in three cancer cells.

PCa progression is mediated by HSP70 and HSP90, which are molecular chaperones, through increasing proliferation and metastasis capacity [53]. The protein levels of HSP70 and HSP90 were decreased in three cells treatment with **1**. Moreover, the mRNA expression level of HSP90 was suppressed in PC-3 and 22Rv1. In addition, the mRNA expression level of HSP70 was suppressed treatment with **1** in C4-2. This study shows that compound **1** inhibits the expression levels of various oncogenes in three types of PCa cells through a variety of suppressed mechanisms.

AR has an important role in cell growth, and cell survival in normal PCa; however, mutations in the AR can contribute to PCa cell progression and therapy resistance. Current PCa treatment approaches inhibit proliferation and metastasis by suppressing AR [54]. 22Rv1 is mutated in AR, C4-2 is derived from androgen-dependent human LNCaP and PC-3 is an androgen-independent cancer cell type. We demonstrated that **1** decreased the mRNA expression level of AR in C4-2 cells while **1** did not affect the mRNA expression level of AR in 22RV1. Therefore, the effect of **1** might not be dependent on AR, **1** might suppress proliferation and metastasis in PCa by disrupting other key cancer signaling pathways like mTOR/AKT and STAT3.

## Conclusion

Overall, this study shows that an investigation of bioactive secondary metabolites from marine *Vibrio* species, strain DJA11 which led to the isolation of compound **1**. Compound **1** significantly suppressed cell viability, invasion, and cell growth. In addition, the protein levels of p-AKT, p-mTOR, p-STAT3, HSP90, and HSP70 were decreased in treatment with compound **1** in three PCa cells. Further studies are needed to identify adjuvant therapy strategies, such as mTOR inhibitors (*e.g.*, rapamycin) or AKT inhibitors, to enhance therapeutic efficacy in PCa therapy.

## Supplemental Materials

Supplementary data for this paper are available on-line only at http://jmb.or.kr.
