# Supplementary material for: 7,7-Bis(3-Indolyl)-p-Cresol, a Metabolite from Marine-Derived Bacterium Vibrio spp. DJA11, Suppresses the Proliferation and Motility of Prostate Cancer Cells
Source: J Microbiol Biotechnol. 2025 May 15;35:e2502035. doi: 10.4014/jmb.2502.02035 (PMC12099627; doi:10.4014/jmb.2502.02035)
Supplement: Supplementary file 1 [file jmb-35-e2502035-supple.pdf]

Supplementary Information

7,7-Bis(3-indolyl)-*p*-cresol, Produced by a Marine-derived Bacterium *Vibrio* spp. DJA11, Suppresses the Proliferation and Motility of Prostate Cancer Cells

Sultan Pulat [1,†], Eun-Young Lee [2,†], Grace Choi [4], Yoon-Hee Jung [4], Sang-Jip Nam [2,3,*], and Hangun Kim [1,*]

[1] *College of Pharmacy and Research Institute of Life and Pharmaceutical Sciences, Sunchon National University, Sunchon 57922, Republic of Korea; sultanpulat@s.scnu.ac.kr (S.P); hangunkim@sunchon.ac.kr (H.K)*

[2] *Department of Chemistry and Nanoscience, Ewha Womans University, Seoul 03760, Republic of Korea; younglee0124@naver.com (E.-Y.L); sjnam@ewha.ac.kr (S.-J.N.)*

[3] *Graduate Program in Innovative Biomaterials Convergence, Ewha Womans University, Seoul 03760, Republic of Korea; sjnam@ewha.ac.kr (S.-J.N.)*

[4] *Department of Biomaterial Research, National Marine Biodiversity Institue of Korea, Seocheon 33662, Republic of Korea; gchoi@mabik.re.kr (G.C); tyu49002@mabik.re.kr (Y.H.J)*

[†] These authors contributed equally to this work

Corresponding Author: hangunkim@sunchon.ac.kr, sjnam@ewha.ac.kr

Table of Contents

Figure S1. ^{1}H NMR spectrum of 7,7-Bis(3-indolyl)-*p*-cresol (**1**) in DMSO-d_6 ………………………………………………………………… **S3**

Figure S2. ^{13}C NMR Spectrum of 7,7-Bis(3-indolyl)-*p*-cresol (**1**) in DMSO-d_6 ……………………………………………………………... **S4**

.

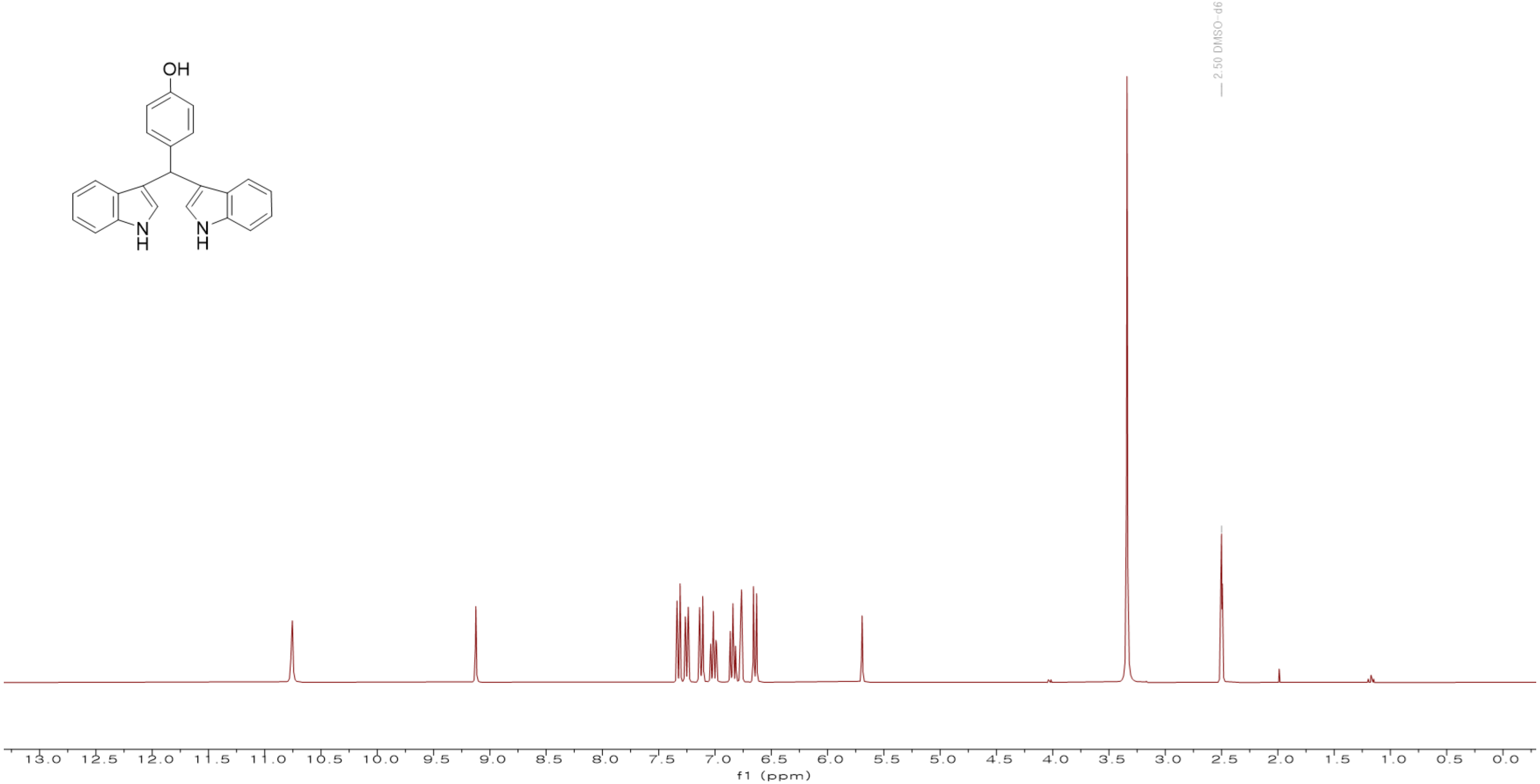

Figure S1. ^{1}H NMR spectrum of 7,7-Bis(3-indolyl)-*p*-cresol (**1**) in DMSO-*d*$_6$

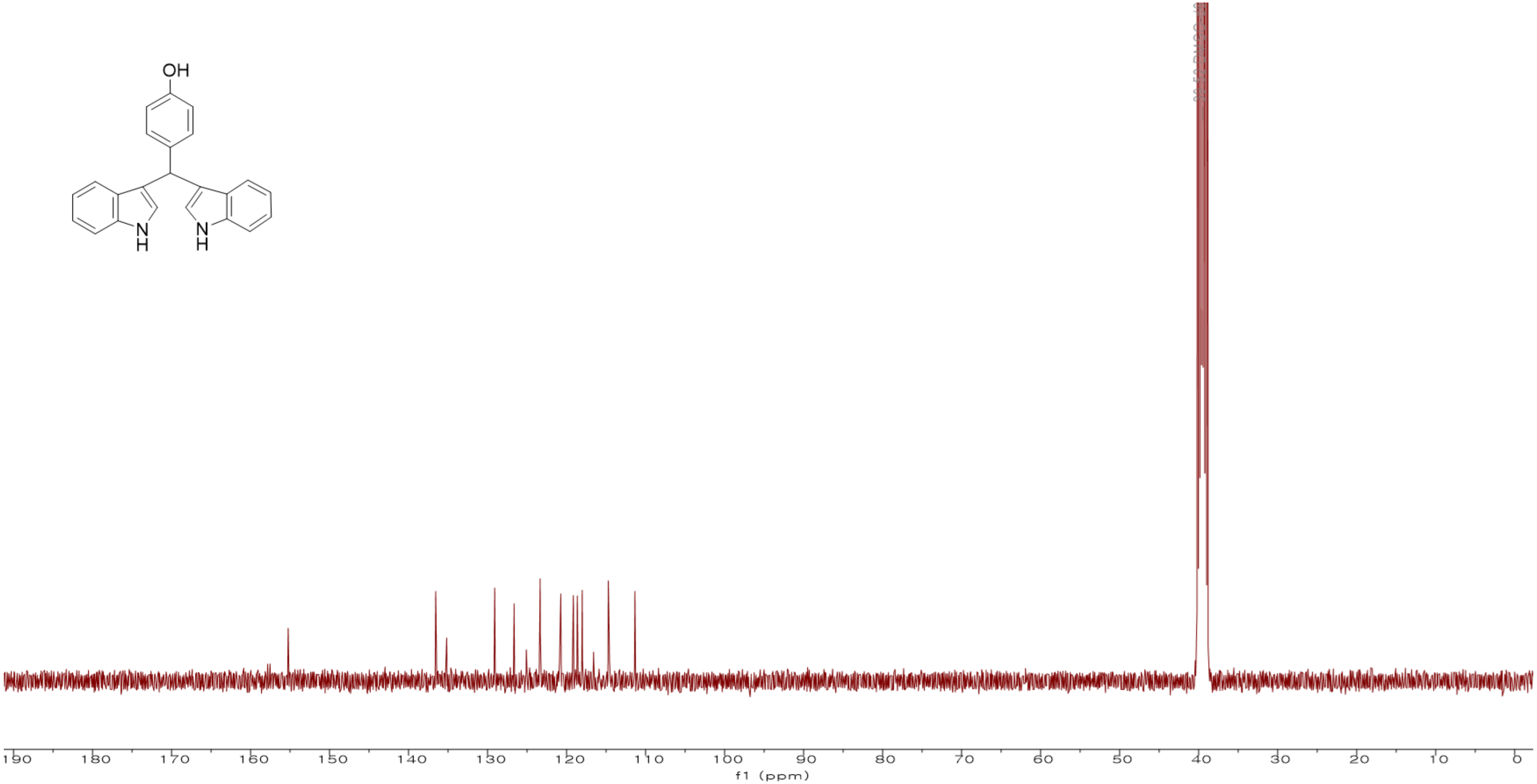

Figure S2. ^{13}C NMR spectrum of 7,7-Bis(3-indolyl)-*p*-cresol (**1**) in DMSO-d_6
